# Prognostic Model of ICU Admission Risk in Patients with COVID-19 Infection Using Machine Learning

**DOI:** 10.3390/diagnostics12092144

**Published:** 2022-09-03

**Authors:** Khandaker Reajul Islam, Jaya Kumar, Toh Leong Tan, Mamun Bin Ibne Reaz, Tawsifur Rahman, Amith Khandakar, Tariq Abbas, Md. Sakib Abrar Hossain, Susu M. Zughaier, Muhammad E. H. Chowdhury

**Affiliations:** 1 Department of Physiology, Faculty of Medicine, University Kebangsaan Malaysia, Kuala Lumpur 56000, Malaysia; 2 Department of Emergency Medicine, Faculty of Medicine, Universiti Kebangsaan Malaysia, Kuala Lumpur 56000, Malaysia; 3 Department of Electrical, Electronics and Systems Engineering, Universiti Kebangsaan Malaysia, Bangi 43600, Malaysia; 4 Department of Electrical Engineering, Qatar University, Doha P.O. Box 2713, Qatar; 5 Urology Division, Surgery Department, Sidra Medicine, Doha P.O. Box 26999, Qatar; 6 Department of Basic Medical Sciences, College of Medicine, QU Health, Qatar University, Doha P.O. Box 2713, Qatar

**Keywords:** intensive care unit, COVID-19, early prediction, machine learning, clinical biomarkers

## Abstract

With the onset of the COVID-19 pandemic, the number of critically sick patients in intensive care units (ICUs) has increased worldwide, putting a burden on ICUs. Early prediction of ICU requirement is crucial for efficient resource management and distribution. Early-prediction scoring systems for critically ill patients using mathematical models are available, but are not generalized for COVID-19 and Non-COVID patients. This study aims to develop a generalized and reliable prognostic model for ICU admission for both COVID-19 and non-COVID-19 patients using best feature combination from the patient data at admission. A retrospective cohort study was conducted on a dataset collected from the pulmonology department of Moscow City State Hospital between 20 April 2020 and 5 June 2020. The dataset contains ten clinical features for 231 patients, of whom 100 patients were transferred to ICU and 131 were stable (non-ICU) patients. There were 156 COVID positive patients and 75 non-COVID patients. Different feature selection techniques were investigated, and a stacking machine learning model was proposed and compared with eight different classification algorithms to detect risk of need for ICU admission for both COVID-19 and non-COVID patients combined and COVID patients alone. C-reactive protein (CRP), chest computed tomography (CT), lung tissue affected (%), age, admission to hospital, and fibrinogen parameters at hospital admission were found to be important features for ICU-requirement risk prediction. The best performance was produced by the stacking approach, with weighted precision, sensitivity, F1-score, specificity, and overall accuracy of 84.45%, 84.48%, 83.64%, 84.47%, and 84.48%, respectively, for both types of patients, and 85.34%, 85.35%, 85.11%, 85.34%, and 85.35%, respectively, for COVID-19 patients only. The proposed work can help doctors to improve management through early prediction of the risk of need for ICU admission of patients during the COVID-19 pandemic, as the model can be used for both types of patients.

## 1. Introduction

The COVID-19 pandemic began in Wuhan, China at the end of 2019, and spread quickly throughout the world [1]. Some countries experienced more than one wave of the pandemic. As of 11 July 2022, globally there have been around 560 M confirmed cases and around 6.3 M deaths caused by COVID-19 [2]. This novel coronavirus mostly affects a patient’s lungs, resulting in pneumonia [3]. The majority of individuals have a mild form of the illness, with typical respiratory symptoms [4]. The most frequent clinical signs are fever and cough; however, some patients develop severe illness, which results in intensive care unit (ICU) admission and even death [5]. During the pandemic, along with the non-COVID critically ill patients, COVID-19 patients were also transferred to ICU [6], which resulted in the demand for ICU resources substantially increasing, exceeding the capacity in many healthcare systems [7]. ICUs are costly and time-sensitive resources, and if their capacity is challenged, it can have a major consequence for healthcare services [8]. In addition, as the number of critically ill patients continues to increase, their stays in the ICU have become even longer [9,10], especially during the COVID-19 situation. To solve this issue, some scoring systems have been used for the early identification of ICU requirements. Scoring techniques rely on the identification of prognostic markers associated with the severity of the disease [11]. Similar scoring systems are available for ICU admission risk prediction among non-COVID patients. Analysis of coagulation parameters has been very useful in identifying the severity of non-COVID-related pneumonia [12]. The severity of COVID-19 can also be identified from a similar analysis of blood-profile deviations, including lymphopenia, thrombocytopenia, and coagulopathies, such as prolonged prothrombin time [13,14,15,16]. However, these studies could be improved further by increasing their robustness by training on a larger dataset, and also by providing an approach that could be used for both COVID and non-COVID patients [17]. A model that can be generalized for both types of patients is not available in the literature, to the best of the authors’ knowledge.

The research implication of this study is the development of a novel framework that reliably and accurately helps in calculating the risk of ICU admission among two types (COVID and non-COVID) of patients. The severity scoring systems can be used to predict adverse outcomes for initial triage and treatment [18]. This can help in the efficient management of ICU resources for timely intervention. During the peak of the pandemic, it may even be crucial to predict and manage the ICU requirement beforehand so that potential capacity challenges can be managed. 

The rest of the paper is organized in the following manner: Section 1 is the introduction outlining the motivation for the study, along with the potential research implication. Section 2 summarizes the relevant works in this domain and also summarizes the contribution of this work. Section 3 provides the methodology, which includes the details of the dataset description, statistical analysis, data preprocessing, feature ranking techniques, machine learning (ML) techniques, and performance metrics used in this study. Section 4 provides the results of the investigations followed by a detailed discussion. Finally, the conclusion is provided in Section 5.

## 2. Related Work

Radiological images, such as chest X-rays (CXR) and computed tomography (CT) images, have been extensively used in the stratification of COVID-19 patients [19,20,21,22,23,24]. Poly et al. [25] have performed a systematic review with a meta-analysis of relevant studies to quantify the performance of deep learning algorithms in the automatic stratification of COVID-19 patients using CXR images. It is reported that the deep learning models provided satisfactory results and can be used in the fast screening of COVID-19 patients. However, these models should be further validated using independent unknown test sets. Several studies have reported the usefulness of CT images for COVID-19 detection and severity stratification and in follow-on studies. CXR- or CT-based approaches cannot be used as prognostic models.

Clinical biomarkers from electronic health records have shown great promise in developing a prognostic model and scoring technique as an early predictor of severity or mortality for COVID-19 patients [26,27,28]. Thus, clinical biomarkers can be used as reliable predictors for COVID-19 stratification, severity detection, and mortality risk prediction. Most importantly, these prognostic models can make reliable predictions based on the data at admission. Electronic health records have been utilized in developing machine learning models for predicting the length of stay (LOS) in patients suffering from sepsis and COVID-19 in ICU [29,30,31,32]. Alabbad et al. [33] used a random forest classifier to predict the ICU requirement of COVID-19 patients and estimated their LOS in ICU with an accuracy of 94.16%, using data from King Fahad University Hospital, Saudi Arabia. The Kuwait Progression Indicator (KPI) Score is an ICU admission risk prediction scheme for COVID-19 patients [34], which uses three biomarkers—lactic dehydrogenase (LDH), lymphocytes, and high-sensitivity C-reactive protein (hs-CRP). Yan et al. [35] were able to predict a patient’s mortality with over 90% accuracy 10 days in advance. It was reported that a high LDH level is a crucial indicator for the vast majority of patients who require urgent medical care. However, it does not include any scoring system that could help doctors to objectively stratify individuals at risk. Chowdhury et al. [36] proposed a nomogram scoring system for early mortality prediction for COVID-19 patients using five biomarkers. The proposed model provided the area under the curve (AUC) of 0.961 and 0.991 for the derivation and validation cohort, respectively. However, the model requires some bio-markers that are not routinely monitored in a resource-limited setup [37]. This can pose a restriction on the wide usage of the proposed scoring system. Moreover, this is a mortality prediction system and cannot be used for ICU-admission risk prediction. Similarly, Lorenzoni et al. [38] used data from 25 ICUs from the Veneto ICU network between 28 February 2020 to 4 April 2021 to predict ICU mortality risk among COVID-19 patients. The reported performance was 0.72–0.90 on the test set, while scores of 0.75–0.85 were observed on an external validation set. Magunia et al. [39] developed a model to stratify patient risk and predict ICU survival and outcomes using data collected in a retrospective and prospective manner from different parts of Germany for COVID-19 ICU patients. Age, inflammatory and thrombotic activity, and severity of acute respiratory distress syndrome (ARDS) at ICU admission were reported as strong contributing factors.

Although machine learning approaches have been utilized for ICU admission risk prediction using clinical biomarkers [40,41,42,43,44,45], to the best of our knowledge, no machine learning model has been developed that can be applied for both COVID-19 and non-COVID patients. Identification and prioritization of the patients at high risk are essential for resource planning as well as treatment planning. Patients can be continuously monitored for ICU admission risk during their hospital stay using an easy-to-use and reliable scoring system. This proposed study can help clinicians make early predictions regarding patients at risk of ICU admission; for patients at risk of organ dysfunction, providing early treatment could save their lives. This study proposed a machine-learning-based model that can reliably predict ICU admission risk among both COVID-19 and non-COVID patients using patient data at hospital admission. This work may help in the development of a framework of prognostic models using machine learning approaches, adding to the body of knowledge. 

## 3. Materials and Methods

The methodology adopted in the study is presented in this section. The study attempted to investigate ICU admission risk prediction for (i) both COVID-19 and non-COVID-19 patients and (ii) COVID-19 patients only. For both experiments, the impacts of combined features and individual features were investigated in terms of ICU prediction. Firstly, different imputation techniques were used in a preprocessing step to impute the missing data. This missing data imputation technique is very common in clinical studies.

**Figure 1:**
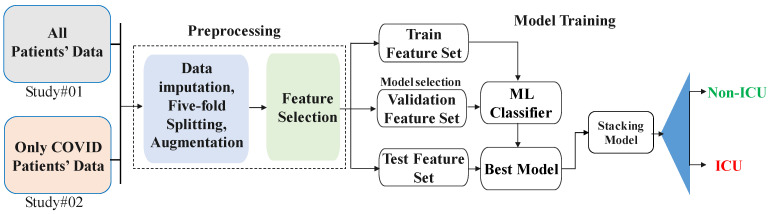
Methodology of the study.

After preprocessing the dataset, three different feature selection methods (Pearson correlation coefficient, chi-square test, and recursive feature elimination) were used for ranking all the parameters in the dataset. Later on, these three techniques were combined to rank the features using the combined scores from three models. A stacking ML model was proposed and compared with the different ML classifiers to predict ICU-requirement risk among COVID-19 and Non-COVID patients. The dataset was divided into training, validation, and testing sets, where the training and validation sets were used to determine the best-performing combination of (i) imputation technique, (ii) combination of features using the feature ranking techniques, and (iii) machine learning model. The testing set was used to state the classification performance. The details of the complete methodology are provided below and can also be referred to in Figure 1.

### 3.1. Study Population

In this study, we used a dataset that contained COVID-19 and non-COVID-19 patients’ data collected from the pulmonology department of Moscow City State Hospital between 20 April 2020 and 5 June 2020 [46]. The dataset contained data from 231 patients; among them, 100 patients were transferred to ICU and 131 patients were non-ICU patients. Of the 231 patients, 156 patients were COVID-19-positive and 75 patients were COVID-19-negative. 

**Figure 2:**
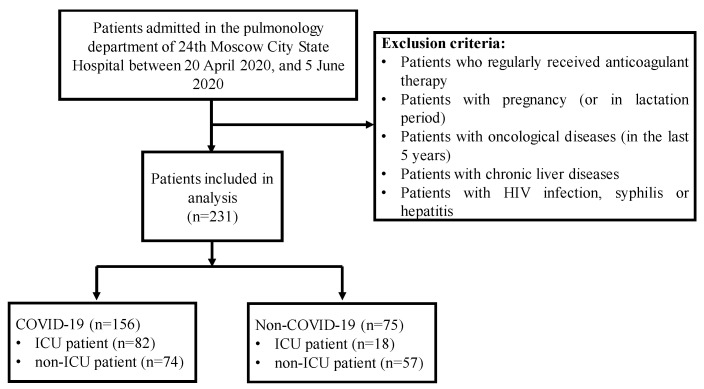
Population details of the study.

Among the COVID-19 patients, confirmed by real-time reverse-transcriptase–polymerase-chain-reaction (RT-PCR) assay of nasal and pharyngeal swab probes, 82 developed refractory respiratory failure (RRF) or severe acute respiratory distress syndrome (SARDS) and were transferred to ICU, and 74 patients had a stable course of the disease and were not transferred to ICU, as shown in Figure 2. Clinical, radiological, and laboratory parameters present upon admission were extracted from electronic health records (EHR). Some of the parameters were present for longitudinal evaluation. However, since all the parameters were not collected longitudinally, longitudinal data could not be considered for the investigation. Only data present at hospital admission were considered. Patients who regularly received anticoagulant therapy before admission, as well as patients with pregnancy (or in lactation period), oncological diseases (in the last 5 years), chronic liver diseases, human immunodeficiency virus (HIV) infection, syphilis, or hepatitis, were excluded from the study.

**Figure 3:**
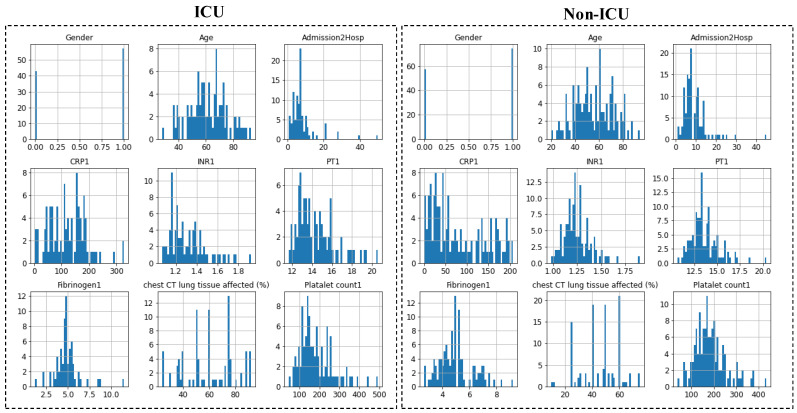
Histogram plots depicting the distributions of nine features at admission among the ICU and non-ICU patients. Here, ‘0′ represents ‘female’ and ‘1′ represents ‘male’.

**Figure 4:**
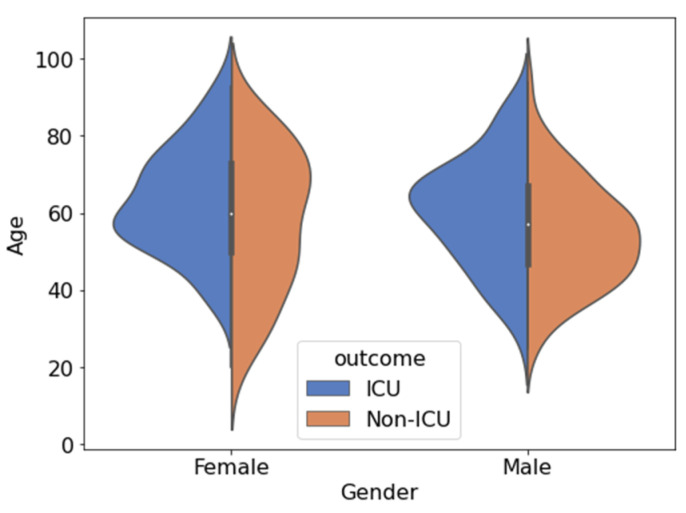
Demographic feature (gender and age) distributions for ICU and non-ICU cases. The blue and orange areas correspond to negative and positive cases, respectively.

Figure 3 shows the distribution of nine features in the dataset: (i) Gender; (ii) Age; (iii) Admission2Hosp (admission to hospital); (iv) CRP (C-Reactive Protein); (v) INR (international normalized ratio); (vi) PT (Prothrombin Time); (vii) Fibrinogen; (viii) Chest CT lung tissue affected, and (ix) Platelet count for ICU and non-ICU patients. The distribution plots confirm that the values for the features were well distributed. For example, the dataset contained patients of all ages, with a higher number of patients in ICU being over 70 years of age while for non-ICU, the average was 60 years. Similarly, CRP among the ICU-patients showed a significantly different distribution compared to that for non-ICU patients. Prothrombin time for the patients varied from 10 to 20 s, with most of the patients having a prothrombin time of around 13 s for both ICU and non-ICU patients. Figure 4 shows the details of demographic variations (age and gender) between the ICU and non-ICU patients. Patients from both categories (ICU and non-ICU patients) are normally distributed among males and females.

### 3.2. Statistical Analysis

Statistical analysis was performed using Python programming language (version 3.7, Creator-Guido van Rossum, The Netherland), where the chi-square univariate test and rank-sum test were conducted to identify the statistically significantly different features among the ICU and Non-ICU (stable) groups. The difference was considered significant if the *p*-value was <0.05. Table 1 summarizes continuous variables, age, and other clinical data reported with a mean and standard deviation of the data, and frequency for each biomarker among the ICU and Non-ICU groups.

### 3.3. Data Preprocessing

#### 3.3.1. Missing Data Imputation

**Figure 5:**
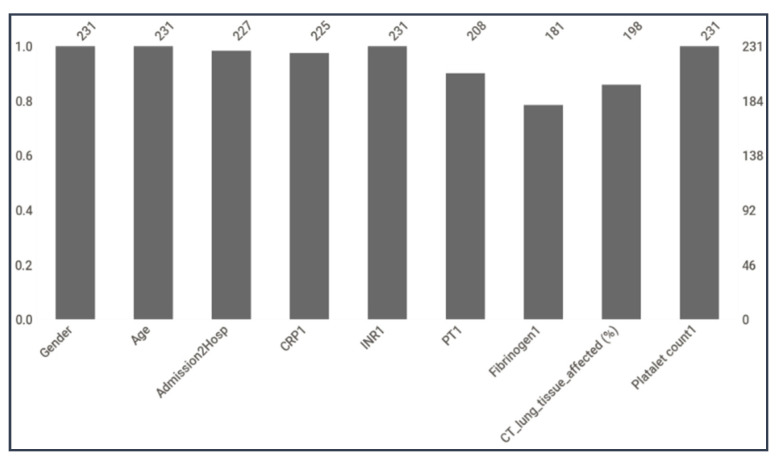
Count of different features in the dataset.

Only the first-day data were used for model training and validation in identifying the primary predictors for ICU admission. Figure 5 shows the count of different features in the dataset, and it can be seen that some parameters were missing for some patients, such as the time between the disease onset and admission to the hospital (days), CRP1, prothrombin time upon admission (PT1 in second), fibrinogen upon admission (Fibrinogen1), and lung tissue affected (%) from chest computed tomography (CT). 

Missing data issues are a constant challenge in clinical data analysis, and this can lead to biased models or degraded model performance. Similarly, a model can produce a biased result if the rows of the missing data are deleted from the study [47]. In this study, three common imputation techniques to tackle the missing data problem were investigated: (i) multiple imputations using the chained equations (MICE) [45], (ii) random forest [48,49], and (iii) nearest neighbor [50] imputation techniques. One of the most common techniques for clinical data imputation is the MICE data imputation technique, which uses logistic regression for binary variables and statistical mean matching for continuous variables. On the other hand, the random forest algorithm has a built-in routine to handle the values that are missing by weighing the frequency of values with the proximity of a random forest after the training of an initially imputed mean dataset. This approach requires a response variable that is useful for random forest training. K-nearest neighbor can predict both discrete attributes (the most frequent value among the k-nearest neighbors) and continuous attributes (the mean among the k-nearest neighbors). The k-nearest neighbor can be easily adapted to work with any attribute as a class, by just modifying which attributes will be considered in the distance metric. 

#### 3.3.2. Balancing the Dataset

An imbalanced dataset can result in a biased model, and therefore, the dataset needs to be balanced. The synthetic minority oversampling technique (SMOTE) is a powerful approach to tackling the imbalance problem [51]. This study investigated two different phases. Firstly, all data were used to predict ICU-admission-risk patients, where ICU and non-ICU patients were 100 and 131, respectively. For the investigation with only COVID-19 patients, ICU and non-ICU patients numbered 82 and 74, respectively. SMOTE technique was used to balance the dataset for different investigations. Rather than using simple replication of minor class data using an over-sampling technique, SMOTE was used to create synthetic data to avoid data imbalance among the classes.

### 3.4. Feature Reduction

**Figure 6:**
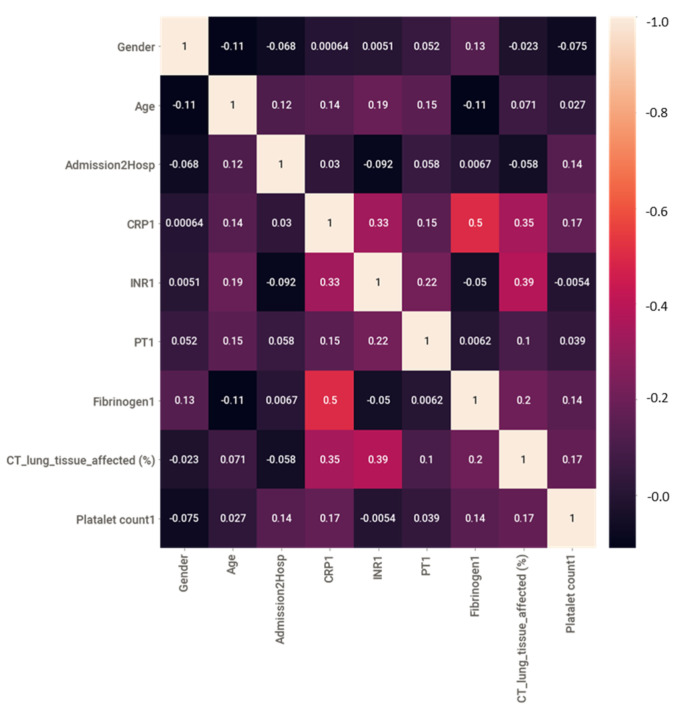
Color map of correlation among different features.

Nine different features were present in the dataset. After preprocessing, the correlation among different features was checked to identify and remove highly correlated features, as removing them has always helped in improving the classifier performance in the author’s previous works [52,53]. As seen in Figure 6, there was no high correlation between the features, and all the features could be used in this study.

### 3.5. Feature Selection

In this study, three different feature selection techniques, namely the chi-square test [54], Pearson correlation coefficient [55], and recursive feature elimination (RFE) [56], were used for detecting ICU admission risk. Additionally, we utilized an average of the feature importance score as a threshold for choosing the features for each technique, after calculating the feature importance score for each feature using each of the three feature selection techniques. Finally, we chose the features for the classification model that exceeded the threshold (feature importance score ≥3) for all three feature selection procedures.

### 3.6. Stacking-Based Machine Learning Model

**Figure 7:**
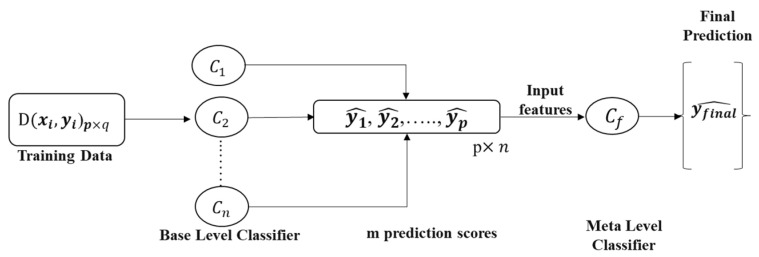
Proposed stacking model architecture.

The study proposed a stacking-based approach and compared the performance with conventional ML classifiers. This approach consisted of a two-step learner comprising a base learner and meta learner; this approach has produced good results in the author’s previous works [20,53,57]. Eight different ML classifiers were investigated in this study, namely random forest [45], support vector machine (SVM) [46], K-nearest neighbor (KNN) [47], XGBoost [48], extra trees [43], gradient boosting [49], MLP [50], and logistic regression [50]. Three feature selection strategies were used to choose the top features, which were then used to compare the different classifiers’ performance. The stacking architecture was used on the three top-performing classifiers ($C_{1},C_{2},C_{3})$ as the base-learner model, and logistic regression was used as the meta-learner model ($C_{f})$ in the second phase of the stacking model to provide the best performance. Figure 7 shows the architecture of the proposed stacking model, which combines n numbers of best-performing classifiers $C,\ldots \ldots ,C_{n}$ using an input dataset D, which had a feature vector ($x_{i}$) and corresponding label ($y_{i}$). In the first step, N base level ML classifier produced the prediction probabilities $y_{1},\ldots \ldots ,y_{p}$. Finally, the prediction probabilities of the best performing base learners fed to a logistic regression-based meta-learner classifier ($C_{f})$ for the final prediction.

### 3.7. Development and Validation of Classification Model

In this study, various machine learning models were examined using 5-fold cross-validation, where 80% of the subjects’ data were used in training and validation and 20% were used in the testing set for a single fold. This was repeated 5-fold, with a new test set on each fold. Several performance criteria, such as sensitivity, specificity, precision, accuracy, and F1-score, were used to assess the performance of several models on the test dataset. Mathematical representations of the different metrics are shown in Equations (1)–(5). The areas under the curve (AUC) for individual predictors, as well as combinations of them, were assessed to ascertain how well-ranked parameters performed in stratifying ICU and non-ICU patients. The performances of unseen (test) folds were combined to create the overall confusion matrix for the 5-fold cross-validation. It is worth noting that leave-one-out cross-validation (LOOCV) was also investigated, where all the subjects apart from one were used for training and validation while one subject’s data were used in testing, and the procedure was repeated based on the number of subjects in the experiment.

(1) $$\mathrm{Accuracy}=\frac{\left(\mathrm{TP}+\mathrm{TN}\right)}{\left(\mathrm{TP}+\mathrm{FN}\right)+\left(\mathrm{FP}+\mathrm{TN}\right)}$$

(2) $$\mathrm{Sensitivity}=\frac{\left(\mathrm{TP}\right)}{\left(\mathrm{TP}+\mathrm{FN}\right)}$$

(3) $$\mathrm{Specificity}=\frac{\left(\mathrm{TN}\right)}{\left(\mathrm{FP}+\mathrm{TN}\right)}$$

(4) $$\mathrm{Precision}=\frac{\left(\mathrm{TP}\right)}{\left(\mathrm{TP}+\mathrm{FP}\right)}$$

(5) $$F1\;\mathrm{Score}=\frac{\left(2*\mathrm{TP}\right)}{\left(2*\mathrm{TP}+\mathrm{FN}+\mathrm{FP}\right)}$$

The number of patients with ICU outcomes classified as ICU is denoted as True Positive (TP), the number of non-ICU patients identified as Non-ICU is denoted as True Negative (TN), the number of non-ICU patients incorrectly identified as ICU is denoted as False Positive (FP), and the number of ICU patients incorrectly identified as Non-ICU is denoted as False Negative (FN). The performance of the ML classifier was assessed using different evaluation metrics with 95% confidence intervals (Cis), calculated using Equation (6).

*r* = *z*√*metr*(1 − *metric*)/*N* (6)

where, *N* is the number of test samples and *z* is the level of significance that is 1.96 for 95% CI. 

## 4. Results

### 4.1. Characteristics and Outcomes

Two different investigations were conducted in this study: (i) an investigation of the ICU admission risk of all patients (*n* = 231), where 156 patients were COVID-19-positive and 75 were COVID-19-negative, and (ii) an investigation of the ICU admission risk of COVID-19-positive (*n* = 156) patients alone. Each investigation was used to identify the best feature combination and individual best feature for detecting ICU admission risk using eight different machine learning classifiers. Three different imputation techniques were used in this study, where the MICE data imputation technique outperformed KNN and random forest for identifying the best feature combination from the ranked features in both investigations. It was found that age, gender, admission-to-hospital time, CRP, fibrinogen, chest CT lung tissue affected (%), and PT had statistically significant differences between ICU and non-ICU groups, while differences between INR and platelet count were statistically insignificant across the groups (Table 1).

### 4.2. Best Feature Combination for Early Prediction of ICU

In both experiments, three different feature selection approaches were used to select a feature combination supported by all these techniques. All of the three feature selection techniques selected five features—CRP, chest CT lung tissue affected (%), age, admission to hospital, and fibrinogen to classify ICU and non-ICU patients, as shown in Table 2.

### 4.3. Development and Validation of the Stacking Model

**Figure 8:**
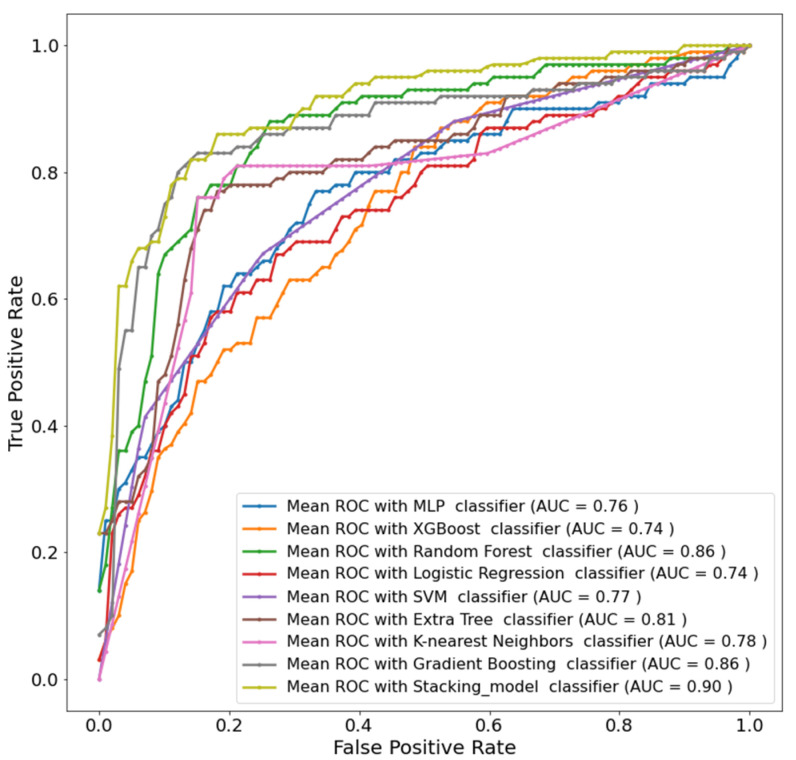
ROC curves using all (both COVID-19 and non-COVID-19) patients’ data.

The five selected features were used in both the investigations using the eight different ML classifiers, and then the stacking approach was implemented to boost the performance further. A logistic regression model was used as a meta-learner in the stacking model. The top three performing classifiers were random forest, gradient boosting, and extra trees. The accuracies obtained from these models were 82.33%, 81.03%, and 79.74% respectively, as shown in Table 3. These three models were used to train the meta-learners logistic regression classifier, which boosted the performance and provided weighted precision, sensitivity, F1-score, specificity, and accuracy of about 84.45%, 84.48%, 83.64%, 84.47%, and 84.48%, respectively. It is evident from Table 3 that the stacking approach improved the accuracy by more than 2%. With 90% AUC, the stacking approach clearly outperforms other ML classifiers, as can be seen in Figure 8.

**Figure 9:**
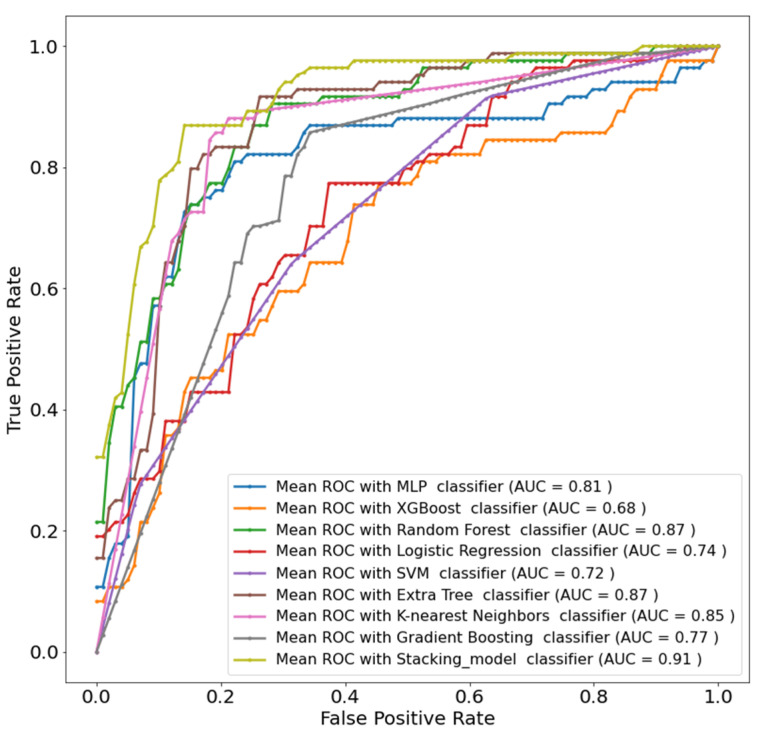
ROC curves using only COVID-19 patient data.

Similarly, for COVID-19 patients only, random forest, extra trees, and K-nearest neighbors were the top three performing classifiers. The accuracies obtained from these models were 83.44%, 82.8%, and 82.17%, as shown in Table 4. The stacking model employed a logistic regression model as a meta-learner. This approach boosted the performance to weighted precision, sensitivity, F1-score, specificity, and overall accuracy of 85.34%, 85.35%, 85.11%, 85.34%, and 85.35%, respectively. Table 4 shows an overall improvement in accuracy of 2% using the stacking model. With 91% AUC, the stacking approach clearly outperforms other ML classifiers, as can be seen in Figure 9.

**Figure 10:**
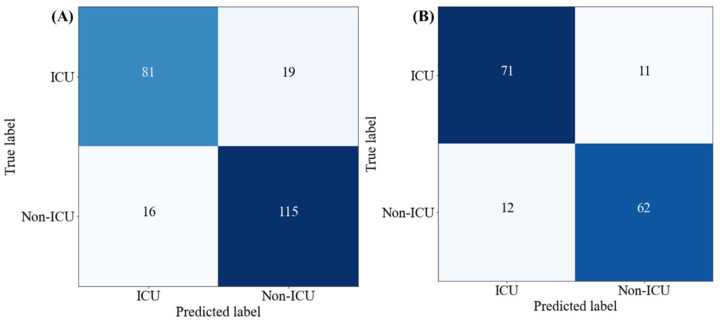
Confusion matrix for ICU and Non-ICU classification using the best performing stacking model for (**A**) all (both COVID-19 and non-COVID-19) patients’ data, and (**B**) only COVID-19 patient data.

Figure 10 shows the confusion matrix for the best-performing stacking model to identify ICU risk patients using all (both COVID-19 and non-COVID-19) patients’ data and using only COVID-19 patients’ data. In Figure 10A it is clearly shown that the stacking model correctly identified 81 out of 100 ICU patients from both COVID-19 and non-COVID-19 patients, while the stacking model correctly identified 71 out of 82 COVID-19 ICU patients (Figure 10B).

### 4.4. Individual Feature as ICU Admission Predictor

The study also investigated individual feature performance with the best-performing stacking classification model to ascertain the top individual feature simultaneously for all (both COVID-19 and non-COVID-19) patients and only COVID-19 patients.

For both COVID-19 and non-COVID-19 patients, chest CT lung tissue affected (%) is the most important feature for the stacking classifier. Table 5A provides the performance metrics with a 95% confidence interval to identify the contribution of the individual features. Chest CT lung tissue affected (%) produced the best performance with overall accuracy and weighted precision, sensitivity, specificity, and F1-score of 74.43%, 77.65%, 74.43%, 74.43%, and 71.75%, respectively.

By contrast, for COVID-19 patients, the time between the disease onset and admission to the hospital (days) was the most important feature for the stacking classifier. Table 5B shows the overall accuracies and weighted average performance with a 95% confidence interval for the other matrices to identify the contribution of individual features using all features for five-fold cross-validation with the best performing classifier. The time between the disease onset and admission to the hospital (days) produced the best performance, with overall accuracy, weighted precision, sensitivity, specificity, and F1-score of 73.9%, 73.99%, 73.9%, 73.9%, and 73.92%, respectively. We also developed and validated all ML models and stacking approaches with the leave-one-out cross-validation (LOOCV) approach, and compared the performances. Supplementary Tables S1 and S2 represent the performances of all models for the two different studies using the LOOCV approach. The stacking approach produced the best performance for both studies, which was comparable with the five-fold CV (see Table 3 and Table 4 for five-fold CV results). However, the experimental time for the stacking model with the LOOCV technique was 25–30 times longer than the five-fold CV technique.

## 5. Discussion

The primary goal of the present study was to accurately identify the ICU admission risk of the hospital patients on the first day of hospital admission using a few clinical parameters. In addition to this, the correlation between ICU admission risk and clinical data for both COVID-19-positive and negative patients and COVID-19-positive patients alone was also investigated. Based on all patients’ data, it was found that five important features (chest CT lung tissue affected (%), CRP, fibrinogen, age, and admission-to-hospital time) in combination performed better than others, and produced 84.48% sensitivity using the stacking model. Similarly, for COVID-19-positive patients alone, it was found that these five features combined using the stacking model outperformed other ML classifiers and produced 85.35% sensitivity. It is evident from this study that for all data or data from COVID patients alone, carefully selected multiple parameters can provide better sensitivity in predicting the ICU admission risk. 

In addition, the most important individual feature for detecting ICU admission risk using combined and COVID-19 patients’ data was also investigated. Based on all patients’ data, it was found that the most impactful feature was the chest CT lung tissue affected (%) which individually can predict ICU patients with 74.43% sensitivity. In [46], it was reported that prothrombin time is a good predictor of ICU admission risk; however, in this study, it was found that PT was a weak predictor, with 57.91% prediction sensitivity for the combined data. For COVID-19-positive patients alone, the time between the disease onset and admission to the hospital (days) was the most impactful feature, individually predicting ICU admission risk with 73.9% sensitivity, while CRP predicted with 64.29% sensitivity. As reported in [46], PT is a good predictor, but this was not observed in this study. Therefore, it is evident that machine-learning-based study can provide better prediction accuracy in comparison to standard statistical analysis. Moreover, the parameters found to be strong contributors in this machine-learning-based study were also reported in other recent studies as strong contributors for COVID mortality prediction, which is highly related to ICU admission risk prediction. 

Age is one of the most commonly agreed risk factors for predicting the outcomes and severity of COVID-19, where patients older than 60 years of age have the highest fatality rates [58]. This is related to the associated risk factors in this particular age group, including renal disease, coronary heart disease/cerebrovascular disease, hypertension, diabetes, low immunity, and previous respiratory disease. Likewise, patients with older age (>60 years) and comorbidity had worse outcomes in severe acute respiratory syndrome (SARS) [59]. However, in this dataset, this trend was not evident, which could be because this dataset was small and the ICU patient group had a widely distributed age range. If this model can be validated on a large population, the effect of age will be pronounced, and the model performance could be further enhanced. 

Several reports have shown a trend toward abnormal hemostasis and coagulation profile in patients with COVID-19 [5]. Hospitalized patients with particularly higher levels of fibrinogen and D-dimer typically develop disseminated intravascular coagulation and lung embolism, and also have worse outcomes [14,60]. Higher serum fibrinogen levels were noted in patients in the early stages of acute respiratory distress syndrome (ARDS). However, these factors were typically explored after hospitalization and were not used as early predictors to determine the patients with potentially severe forms of COVID-19 based on their initial presentation [61]. Hospitalized patients with severe COVID 19 show a trend toward hyperfibrinolysis and loss of coagulation factor (fibrinogen). Furthermore, fibrinogen is an acute phase reactant that increases during infection and provokes hypercoagulation. It is a glycoprotein that covers fibrin when exposed to thrombin leads to clot formation to stop bleeding. Zou et al. [30] concluded that fibrinogen greater than 7.0 g/L is more prevalent in patients with severe COVID-19 disease (around 19.1%) compared to patients with mild COVID-19 disease (around 5.7%). At later stages of the disease, fibrinogen levels decrease, and fibrinogen less than 2.0 g/L is considered an indicator of thromboprophylaxis and cause for hospital admission [62]. In the current study, since we used the data at admission, most of the patients were not severe at that time, and therefore, while fibrinogen was found to be a useful predictor, it was not the best predictor. However, if we had had longitudinal data, we could have evaluated the model performance when the patients were admitted to ICU.

Serum C-reactive protein (CRP) is a critical indicator that changes considerably in patients with severe COVID-19 [30]. CRP is an early marker of inflammation and infection and is produced by the liver [63]. CRP binds to phosphocholine, which is expressed on the surfaces of damaged cells and which modulates phagocytic activities [64]. It was found that CRP changes significantly at the early stages of the disease in patients with COVID-19, as reported previously [15], and patients who died from the disease had levels of serum CRP 10 times greater in comparison to those who recovered [16]. This is in line with the finding of this study. In both of the investigations, CRP was found to be an important biomarker.

Chest CT is a widely available and noninvasive modality for pneumonia detection and monitoring. When RT-PCR was utilized as the gold standard tool, CT chest was shown to have 97% sensitivity and 68% accuracy in diagnosing COVID-19 [14]. Furthermore, follow-up CT chest showed that 42% of the patients had their lung abnormalities resolved before RT-PCR became negative [14]. Furthermore, CT chest is effective in diagnosing COVID in the absence of symptoms, and is considered an early marker for possible worsening of disease severity [65]. Likewise, changes in CT findings were shown to be an early predictor for speeding up the diagnostic workup in symptomatic patients [66]. The typical findings include bilateral, multi-lobe, posterior peripheral ground-glass opacities, as defined by the Radiological Society of North America (RSNA) Consensus statement [67]. Several CT severity scoring systems are valid for evaluating disease severity and burden [68,69]. It is evident from the above discussion that lung infection manifestation is a vital parameter for later-stage COVID-19 infection detection and quantification. However, in the early infection stage, it could be a good predictor for COVID-19 patients, but not the best predictor.

Predicting the ICU risk for patients when they are admitted to the hospital can greatly aid the hospital management team in allocating the proper resources to the appropriate patient during a crisis. Ineffective management of resources and distribution during the early stages of the pandemic in many countries has led to extremely high patient mortality rates as no ML-based prediction tool was used. In contrast, patients who do not require ICU admission but who are at risk can receive care in the special ward, lessening the strain on hospitals and healthcare facilities. The proposed model is deployed as a web application that can be used by clinicians. The details of the deployment are outside of the scope of this work; however, the link (https://qu-mlg.com/projects/qu-ukm-icucare, accessed on 1 March 2022) is shared here, so that clinicians and interested readers can use it.

The limitation of the proposed tool is that it takes into consideration several clinical and biological parameters and does not integrate symptoms, vitals, and treatments, and therefore, has a risk of bias. The model presented in this work utilizes several clinical variables that can be acquired in most clinics and hospitals, making this work potentially suitable for deployment in a wide range of patient evaluations. However, some further useful biomarkers (e.g., procalcitonin, D-dimers, neutrophils, lymphocytes, etc.) could be investigated, as these were not present in this dataset. Lastly, the number of patients studied was not high, which may cause the developed model to be less generalizable. The overall model performance and generalizability could be significantly improved if these concerns could be resolved.

## 6. Conclusions

In conclusion, the proposed model in this study can predict the ICU admission risk for patients with good discrimination for COVID-19 or non-COVID patients, with 90% AUC, and with 91% AUC for COVID-19 cases alone. Five predictors (chest CT lung tissue affected (%), CRP, fibrinogen, age, and admission-to-hospital time) were required for both studies. The model can predict the risk of admission to ICU based on the hospital admission data; i.e., predicting it much earlier than the real clinical outcome. This study evaluated the various combinations of feature selection approaches, features, and machine learning classifiers. Classical machine learning classifiers are computationally inexpensive and easy to deploy [52], while they can provide better performance in tabular values, such as the data from electronic health records (EHRs). Thus, the proposed study can help physicians in patient stratification in the early stages, which will ultimately facilitate better and more efficient resource management and thereby lessen strain on healthcare resources, reducing mortality risk by supporting seriously ill patients earlier. The proposed framework is deployed as a web application, which can be easily used by clinicians. Considering the potential of this application for pandemic/non-pandemic situations, the authors plan to collect more patient data from the Hamad General Hospital (HGH) to make the model more generalized, and to externally validate the model on a larger dataset. The authors will also continue to work on making the model robust and suitable for a different population.

## Figures and Tables

**Table 1:** Statistical characteristic analysis of all patients in ICU and Non-ICU groups using the dataset.

Item	ICU	Non-ICU	Total	Method	χ^2^ = 17.5	*p* Value
Gender				Chi-square test	χ2 = 17.5	<0.05 *
Male (%)	57 (57%)	74 (56.5%)	131 (57%)			
Female (%)	43 (43%)	57 (43.5%)	100 (43%)			
Age (years)				Rank-sum test	Z = −6.2	<0.05 *
N(missing)	100 (0)	131 (0)	231 (0)			
Mean ± SD	61.6 ± 13.8	55.9 ± 15.7	58.4 ± 15.1			
Q1, Q3	53, 70.2	44, 68.5	48.0, 69.0			
Min, Max	28, 93	20, 94	20, 94			
The time between the disease and admission to hospital (Admission 2Hospital) (Days)				Rank-sum test	Z = −6.2	<0.05 *
N(missing)	99 (1)	128 (3)	227 (4)			
Mean ± SD	7.9 ± 7.45	9.1 ± 5.68	8.6 ± 6.53			
Q1, Q3	4, 8	6, 11	5.0, 10.0			
Min, Max	1, 50	1, 45	1, 50			
C-reactive protein 1(CRP1) (mg/L)				Rank-sum test	Z = −4.34	<0.05 *
N(missing)	97 (3)	128 (3)	225 (6)			
Mean ± SD	123 ± 67.1	78 ± 61.7	97 ± 67.9			
Q1, Q3	64, 166	26, 134	40, 157			
Min, Max	4, 328	1, 207	1, 328			
International normalized ratio (INR)				Rank-sum test	Z = 6.53	0.78
N(missing)	86 (14)	122 (9)	208 (23)			
Mean ± SD	1.32 ±0.18	1.25 ± 0.14	1.28 ± 0.16			
Q1, Q3	1.17, 1.4	1.16, 1.3	1.17, 1.36			
Min, max	1.07,1.92	0.98, 1.9	0.98, 1.92			
Prothrombin time 1 (PT1) (s)				Rank-sum test	Z = 3.27	<0.05 *
N(missing)	86 (14)	122 (9)	208 (23)			
Mean ± SD	14.39 ± 1.91	13.63 ± 1.52	13.95 ± 1.73			
Q1, Q3	12.9, 15.3	12.7, 14.2	12.8, 14.9			
Min, max	11.7, 20.6	10.7, 20.4	10.7, 20.6			
Fibrinogen 1 (mg/L)				Rank-sum test	Z = −5.89	<0.05 *
N(missing)	68 (32)	113 (18)	181 (50)			
Mean ± SD	4.9 ± 1.44	4.99 ± 1.25	4.9 ± 1.32			
Q1, Q3	4.2, 5.3	4.18, 5.45	4.2, 5.4			
Min, max	1.2, 11.5	2.68, 9.21	1.2, 11.5			
Chest CT lung tissue affected (%)				Rank-sum test	Z = −1.11	<0.05 *
N(missing)	88 (12)	110 (21)	198 (33)			
Mean ± SD	59.9 ± 19	46.1 ± 14.1	52.2 ± 17.8			
Q1, Q3	49.5, 75	40, 60	40, 60			
Min, max	24, 92	10, 75	10, 92			
Platelet count 1 ($10^{9}$/L)				Rank-sum test	Z = 4.74	0.44
N(missing)	100 (0)	131 (0)	231 (0)			
Mean ± SD	182 ± 83.2	183 ± 68.8	183 ± 75.2			
Q1, Q3	126, 233	138, 216	129, 219			
Min, max	47, 493	38, 436	38, 493			
Outcome	100 (43%)	131 (57%)	231 (100%)			

* *p* value less than 0.05 is significant.

**Table 2:** Ranked Features by different algorithms.

Features	Pearson Correlation Coefficient	Chi-Square Test	Recursive Feature Elimination	Total
CRP	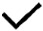	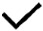	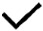	3
Chest CT lung tissue affected (%)	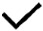	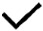	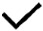	3
Age	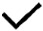	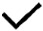	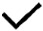	3
Admission2Hospital	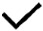	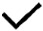	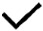	3
Fibrinogen	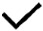	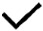	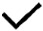	3
Platelet Count	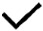	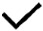		2
Gender		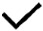	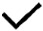	2
PT	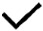		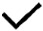	2
INR	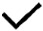		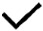	2

**Table 3:** Performance comparison between different ML classifiers using all (both COVID-19 and non-COVID-19) patients’ data.

Classifier	Overall	Weighted with 95% CI			
	Accuracy	Precision	Sensitivity	Specificity	F1-Score
Support Vector Machine (SVM)	61.21 ± 1.99	63.17 ± 1.97	61.21 ± 1.99	63.09 ± 1.97	61.29 ± 1.99
XGBoost (XGB)	65.52 ± 1.94	65.92 ± 1.93	65.52 ± 1.94	65.15 ± 1.94	65.64 ± 1.94
MLP	71.12 ± 1.85	70.98 ± 1.85	71.12 ± 1.85	69.4 ± 1.88	71.02 ± 1.85
Logistic Regression (LR)	71.12 ± 1.85	70.92 ± 1.85	71.12 ± 1.85	68.67 ± 1.89	70.86 ± 1.85
K-Nearest Neighbors (KNN)	71.55 ± 1.84	71.55 ± 1.84	71.55 ± 1.84	70.45 ± 1.86	71.55 ± 1.84
Extra Trees (ET)	79.74 ± 1.64	79.68 ± 1.64	79.74 ± 1.64	78.11 ± 1.69	79.64 ± 1.64
Gradient Boosting (GB)	81.03 ± 1.6	80.98 ± 1.6	81.04 ± 1.6	79.81 ± 1.64	80.98 ± 1.6
Random Forest (RF)	82.33 ± 1.56	82.33 ± 1.56	82.33 ± 1.56	80.55 ± 1.61	82.2 ± 1.56
**Stacking model (RF+ GB+ ET)**	**84.48 ± 1.48**	**84.45 ± 1.48**	**84.48 ± 1.48**	**83.64 ± 1.51**	**84.47 ± 1.48**

**Table 4:** Performance comparison between different ML classifiers using only COVID-19 patient data.

Classifier	Overall	Weighted with 95% CI			
	Accuracy	Precision	Sensitivity	Specificity	F1-Score
XGBoost (XGB)	67.52 ± 2.32	67.86 ± 2.32	67.52 ± 2.32	67.82 ± 2.32	67.56 ± 2.32
Support Vector Machine (SVM)	71.97 ± 2.23	72.4 ± 2.22	71.97 ± 2.23	72.42 ± 2.22	72 ± 2.23
MLP	77.71 ± 2.07	77.92 ± 2.06	77.71 ± 2.07	77.93 ± 2.06	77.73 ± 2.06
Gradient Boosting (GB)	78.98 ± 2.02	79.34 ± 2.01	78.98 ± 2.02	79.4 ± 2.01	79 ± 2.02
Logistic Regression (LR)	80.25 ± 1.98	80.25 ± 1.98	80.25 ± 1.98	79.97 ± 1.99	80.25 ± 1.98
K-Nearest Neighbors (KNN)	82.17 ± 1.9	82.26 ± 1.9	82.16 ± 1.9	81.45 ± 1.93	82.09 ± 1.9
Extra Tree (ET)	82.80 ± 1.87	82.90 ± 1.87	82.80 ± 1.87	82.90 ± 1.87	82.82 ± 1.87
Random Forest (RF)	83.44 ± 1.84	83.49 ± 1.84	83.44 ± 1.84	83.45 ± 1.84	83.45 ± 1.84
**Stacking model (RF + ET+ KNN)**	**85.35 ± 1.75**	**85.34 ± 1.76**	**85.35 ± 1.75**	**85.11 ± 1.77**	**85.34 ± 1.76**

**Table 5:** Comparison of performance evaluation parameters to identify the best classifier for individual feature impact to predict ICU admission risk patients using (**A**) both COVID-19 and non-COVID-19 patients’ data, and (**B**) only COVID-19 patients’ data.

	**(A)**				
	**95% Confidence Interval Results**				
**Feature**	**Overall** **Accuracy**	**Weighted** **Precision**	**Weighted** **Recall**	**Weighted** **Specificity**	**Weighted** **F1-Score**
CRP	71.11 ± 1.85	70.56 ± 1.86	71.11 ± 1.85	71.11 ± 1.85	70.58 ± 1.86
**Chest CT lung tissue affected (%)**	**74.43 ± 1.78**	**77.65 ± 1.7**	**74.43 ± 1.78**	**74.43 ± 1.78**	**71.75 ± 1.84**
Age	61.01 ± 1.99	36.46 ± 1.96	61.01 ± 1.99	61.01 ± 1.99	45.34 ± 2.03
Admission2Hospital	62.41 ± 1.98	37.86 ± 1.98	62.41 ± 1.98	62.41 ± 1.98	46.74 ± 2.03
Fibrinogen	65.31 ± 1.98	65.62 ± 1.97	65.31 ± 1.98	65.31 ± 1.98	65.47 ± 2.04
Platelet Count	67.01 ± 1.92	42.46 ± 2.02	67.01 ± 1.92	67.01 ± 1.92	51.34 ± 2.04
Gender	64.21 ± 1.95	61.51 ± 1.98	64.21 ± 1.95	64.21 ± 1.95	62.65 ± 2.04
PT	57.91 ± 1.9	43.36 ± 2.02	57.91 ± 1.9	57.91 ± 1.9	52.24 ± 2.04
INR	59.81 ± 2	35.26 ± 1.95	59.81 ± 2	59.81 ± 2	44.14 ± 2.02
	**(B)**				
	**95% Confidence Interval Results**				
**Feature**	**Overall Accuracy**	**Weighted Precision**	**Weighted** **Recall**	**Weighted** **Specificity**	**Weighted** **F1-Score**
CRP	64.29 ± 2.38	64.6 ± 2.37	64.29 ± 2.38	64.29 ± 2.38	64.29 ± 2.38
Chest CT lung tissue affected (%)	68.77 ± 2.3	71.79 ± 2.23	68.77 ± 2.3	68.77 ± 2.3	67.88 ± 2.32
Age	55.95 ± 2.46	56.51 ± 2.46	55.95 ± 2.46	55.95 ± 2.46	55.75 ± 2.46
**Admission2Hospital**	**73.9 ± 2.18**	**73.99 ± 2.18**	**73.9 ± 2.18**	**73.9 ± 2.18**	**73.92 ± 2.18**
Fibrinogen	57.24 ± 2.46	57.15 ± 2.46	57.24 ± 2.46	57.24 ± 2.46	57.17 ± 2.46
Platelet Count	47.62 ± 2.48	47.96 ± 2.48	47.62 ± 2.48	47.62 ± 2.48	47.2 ± 2.48
Gender	52.75 ± 2.48	53.19 ± 2.48	52.75 ± 2.48	52.75 ± 2.48	52.57 ± 2.48
PT	51.47 ± 2.48	50.64 ± 2.48	51.47 ± 2.48	51.47 ± 2.48	50.43 ± 2.48
INR	53.39 ± 2.48	53.68 ± 2.47	53.39 ± 2.48	53.39 ± 2.48	53.37 ± 2.48

## Data Availability

Data used in this study can be accessed from Baranovskii et al. [46].

## Supplementary Materials

The following supporting information can be downloaded at: https://www.mdpi.com/article/10.3390/diagnostics12092144/s1, Table S1: Performance comparison between different ML classifiers using all (both COVID-19 and non-COVID-19) patients’ data with LOOCV. Table S2: Performance comparison between different ML classifiers using only COVID-19 patient data with LOOCV.
