# Supplementary material for: Prognostic Model of ICU Admission Risk in Patients with COVID-19 Infection Using Machine Learning
Source: Diagnostics (Basel). 2022 Sep 3;12(9):2144. doi: 10.3390/diagnostics12092144 (PMC9498213; doi:10.3390/diagnostics12092144)
Supplement: Supplementary file 1 [file diagnostics-12-02144-s001.zip › diagnostics-1877099-supplementary.pdf]

Supplementary Materials

Supplementary Table S1: Performance comparison between different ML classifiers using all (both COVID-19 and non-COVID-19) patients' data with LOOCV

Classifier	Overall	Weighted with 95% CI			
	Accuracy	Precision	Sensitivity	Specificity	F1-score
Support Vector Machine (SVM)	81.43 ± 1.59	81.69 ± 1.58	81.43 ± 1.59	81.09 ± 1.6	81.51 ± 1.58
XGBoost (XGB)	79.26 ± 1.65	80.47 ± 1.62	79.26 ± 1.65	80.39 ± 1.62	79.39 ± 1.65
MLP	80.13 ± 1.63	80.03 ± 1.63	80.13 ± 1.63	78.68 ± 1.67	80.07 ± 1.63
Logistic Regression (LR)	81.43 ± 1.59	81.8 ± 1.57	81.43 ± 1.59	81.33 ± 1.59	81.53 ± 1.58
K-Nearest Neighbors (KNN)	78.4 ± 1.68	80.91 ± 1.6	78.4 ± 1.68	80.91 ± 1.6	78.4 ± 1.68
Extra Tree (ET)	79.26 ± 1.65	79.58 ± 1.64	79.26 ± 1.65	78.97 ± 1.66	79.36 ± 1.65
Gradient Boosting (GB)	80.56 ± 1.61	80.45 ± 1.62	80.56 ± 1.61	79.01 ± 1.66	80.48 ± 1.62
Random Forest (RF)	81 ± 1.6	80.91 ± 1.6	80.99 ± 1.6	79.58 ± 1.64	80.94 ± 1.6
**Stacking model (RF+ LR+ SVM)**	**83.4 ± 1.55**	**83.97 ± 1.5**	**83.4 ± 1.55**	**81.33 ± 1.74**	**83.62 ± 1.63**

Supplementary Table S2: Performance comparison between different ML classifiers using only COVID-19 patient data with LOOCV

Classifier	Overall	Weighted with 95% CI			
	Accuracy	Precision	Sensitivity	Specificity	F1-score
XGBoost (XGB)	79.97 ± 1.99	79.78 ± 1.99	79.97 ± 1.99	78.08 ± 2.05	79.83 ± 1.99
Support Vector Machine (SVM)	83.43 ± 1.85	83.69 ± 1.83	83.43 ± 1.85	83.09 ± 1.86	83.51 ± 1.84
MLP	81.26 ± 1.94	82.47 ± 1.89	81.26 ± 1.94	82.39 ± 1.89	81.39 ± 1.93
Gradient Boosting (GB)	82.13 ± 1.9	82.03 ± 1.91	82.13 ± 1.9	80.68 ± 1.96	82.07 ± 1.9
Logistic Regression (LR)	83.43 ± 1.85	83.8 ± 1.83	83.43 ± 1.85	83.33 ± 1.85	83.53 ± 1.84
K-Nearest Neighbors (KNN)	80.4 ± 1.97	82.91 ± 1.87	80.4 ± 1.97	82.91 ± 1.87	80.4 ± 1.97
Extra Tree (ET)	81.26 ± 1.94	81.58 ± 1.92	81.26 ± 1.94	80.97 ± 1.95	81.36 ± 1.93
Random Forest (RF)	82.56 ± 1.88	82.45 ± 1.89	82.56 ± 1.88	81.01 ± 1.95	82.48 ± 1.89
**Stacking model (RF+ LR+ SVM)**	**84.1 ± 1.81**	**84.01 ± 1.82**	**84.09 ± 1.82**	**82.68 ± 1.88**	**84.04 ± 1.82**
